# My winding journey to a career in hospital epidemiology: Serendipity, detours and opportunities

**DOI:** 10.1017/ash.2022.378

**Published:** 2023-02-10

**Authors:** Daniel J. Sexton

**Affiliations:** Duke University Medical Center, Durham, North Carolina

The term “hospital epidemiology” was not in my lexicon when I began my career in infectious diseases (ID) in 1977. I recently verified this by performing a PubMed search using the term “hospital epidemiology” for the years 1958 to 1977. It revealed exactly 7 papers! Therefore, it is not surprising that my journey followed a long, winding, and serendipitous path.

I grew up on a farm in northern Iowa as the third of 10 children. My father was a veterinarian. I left high school early and graduated from Loras College, a small liberal arts school in Dubuque, Iowa, 3 years later. I entered Northwestern University Medical School in 1967 with naïve aspirations of becoming a cardiologist. However, that dream faded during my internship at Mount Sinai Hospital in New York City. Exhausted and demoralized by punishingly long hours and continuous exposure to patients with failing organs and untreatable degenerative diseases, I briefly considered becoming a pathologist. However, my career took a fortuitous turn when I was drafted into military service in January 1972. Dr. Sol Berson, the Chief of Medicine at Mount Sinai called me into his office in April 1972 and offered to help me fulfill my military obligation in the US Public Health Service. Three months later, I was at the Centers for Diseases Control and Prevention (CDC) in Atlanta taking a course in epidemiologic methods and principles. In August 1972 I was assigned to the CDC Field Services Branch and was sent to the Mississippi State Health Department in Jackson.

One of my early investigations as an Epidemic Intelligence Service (EIS) Officer focused on a cluster of cases of Rocky Mountain spotted fever (RMSF) near Oxford, Mississippi. I placed a “cold call” to ask Dr. Willy Burgdorfer if he would help me investigate this cluster. Dr. Burgdorfer enthusiastically agreed to provide laboratory assistance. Subsequently, I made 2 trips to the National Institutes of Health Rocky Mountain Laboratory in Hamilton, Montana, where I learned how to identify and dissect ticks, perform serologic testing, and isolate spotted-fever–group rickettsia. This training and Dr. Burgdorfer’s enduring support and encouragement eventually led to over 30 published studies on spotted-fever–group rickettsial infections over the following 20 years.

Influenced by my stint as an EIS officer, I applied for a medical residency in 1974 with the new goal of becoming an infectious diseases (ID) specialist and was accepted as a first-year resident at University of Texas Southwestern Medical Center. However, a few months later I was notified that funding for my residency position had been rescinded. I was without a job 2 months before the start of my residency! Fortunately, Dr. David Smith, a Duke graduate and young ID faculty member at University of Mississippi, came to my rescue by informing me of a last-minute opening in the Duke ID fellowship program. I was accepted and I began my fellowship on July 1, 1975, despite not yet having completed internal medicine residency. As the sole ID fellow, I performed all of the ID consultations at Duke and the Durham VA Hospitals and was on-call 50 weekends of the year. Between consultations and conferences, I collected blood samples and convalescent sera from patients with Rocky Mountain spotted fever that I sent to Dr. Burgdorfer as part of our ongoing research collaboration.

Although I was promised a medicine residency position at Duke if I continued conducting research for another year, I was eager to complete residency without delay. Thus, I left Duke and entered medicine residency at the University of Missouri in Columbia.

I was offered a faculty position in the ID division at Duke 2 years later, with a starting salary of $28,000. I turned it down and instead accepted a position at a multispecialty clinic in Oklahoma City because my wife was pregnant with our second child and we were deeply in debt. Dr. Burgdorfer argued against my decision, but when I persisted, he suggested that we continue to collaborate on RMSF research while I was in private practice.

The next 11 years were hectic. I had a busy consultative ID practice. The AIDS epidemic diverted my interests and consumed enormous amounts of my time, but I continued to collect data on the clinical aspects of RMSF, to collect ticks for various studies, and to write papers with Dr. Burgdorfer and another leading rickettsial investigator, Dr. David Walker.

In the early 1980s I also made initial steps to becoming a hospital epidemiologist by securing paid positions to supervise the Infection Control programs at 3 community hospitals in Oklahoma City. Talented local infection control practitioners taught me the “nuts and bolts” of the surveillance of healthcare-associated infections and existing and evolving rules regarding Joint Commission on Accreditation of Healthcare Organizations (JCAHO) surveys. I read the early and evolving infection control literature and learned how to negotiate with and influence hospital administrators. In the process, I realized that it was possible to form a network focused on infection prevention.

However, 11 years after finishing my training, the long hours and unceasing demands of private practice were wearing me down. My wife and I had anticipated that this might happen and decided that it was time to activate our long-term plan to take a sabbatical in Australia. We had both dreamed about living abroad and had prepared by saving $1,000 per month for 100 consecutive months. I was able to secure an Australian medical license and an unpaid position as a senior lecturer in infectious diseases at Monash University through the help of another former Duke ID fellow, Dr. Malcolm McDonald. We purchased 5 around-the-world airline tickets and arrived in Melbourne in January 1989. Our 3 children enrolled in Australian schools. I audited a course in molecular biology at Monash University, made ID rounds at 2 Melbourne hospitals, taught medical students about clinical medicine and collaborated with new colleagues in studies on Queensland tick typhus and Flinders Island spotted fever.

My former mentor at Duke, Dr. John Hamilton, invited me to return to Duke as an ID faculty member while I was in Australia. I immediately accepted and returned to Duke in January 1990. My career change resulted in a substantial drop in income, but I was able to supplement this by doing consultations and supervising the infection control program at Durham Regional Hospital. Although I was expected to do clinical service in ID and general medicine full time, I pursued and expanded my interest in rickettsial infections in my “spare time.” Two years later, I wrote a business plan and convinced the Duke administration to provide me a reasonable salary to take over the infection control program at Duke and chair the Duke Hospital Infection Control Committee. In turn, this allowed me to reduce my clinical duties while increasing my time for research.

Dr. Eugene Stead, Jr, the former Chair of Medicine and Emeritus Professor at Duke, moved into an office next to mine in 1995. Dr. Stead and I connected immediately; he convinced me to pursue community-based research, an “arena” that most investigators had totally ignored.

In 1997 I asked the Duke Private Diagnostic Clinic to loan me $100,000 to develop a network of infection control programs in community hospitals in central North Carolina. I signed a note to pay the loan back in 2 years. I hired my first “outreach” infection control preventionist and negotiated contracts with 4 community hospitals—the “Duke Infection Control Outreach Network (DICON)” was launched. One year later, I paid off my loan, recruited several additional community hospitals to join my embryonic network, hired a second nurse, and began a rudimentary database on healthcare-associated infections in community hospitals. In 2005, I hired Paul Thacker to become the administrator for DICON, and Dr. Deverick Anderson joined me in the day-to-day operations of DICON following completion of his ID fellowship at Duke. During the ensuing years, >10 talented epidemiologists joined our team, including Rebekah Moerhing, Arthur Baker, Chris Hostler, Becky Smith, and Libby Dobbs-Ashley.

As we celebrate its 25th anniversary this year, DICON has grown to include >60 hospitals and surgical centers in 6 southeastern states. DICON has served as the foundation for the continuing growth of our entire program. Our research expanded rapidly after we joined the CDC Prevention Epicenters Program. In 2013 we formed the Duke Antimicrobial Stewardship Outreach Network (DASON). In 2016 we merged DICON, DASON, the Duke Hospital Epidemiology and Antimicrobial Stewardship programs, our ID/Infection Control Fellowship and our outreach educational programs into the Duke Center for Antimicrobial Stewardship and Infection Prevention. At present, the center has >50 full-time employees including 7 liaison infection control practitioners, 5 liaison pharmacists, 11 hospital epidemiologists, and 2 ID fellows, several study coordinators and information technology specialists; its own disinfection, resistance, and transmission epidemiology laboratory; ongoing research support from the CDC and National Institutes of Health; and outreach education and online training programs for hospitals within and outside DICON and DASON. Several central databases contain data on >2.4 million surgical procedures, 25,000 surgical site infections, 42 million patient days, and 1.4 million admissions during the past 9 years. The DASON central database has detailed antimicrobial prescribing data on 32 million doses of antimicrobial agents and >23 million patient days for the same period.

## Lessons learned

Although my career in hospital epidemiology involved serendipity, it was possible because I had excellent training early in my career in research and clinical medicine. Over time I developed further experience that helped me and my colleagues explore answers to previously unanswered questions and devise better ways to apply hospital epidemiologic principles within community hospitals. I was fortunate to have mentors and teachers like Willy Burgdorfer, John Hamilton, Eugene Stead, and Sol Berson, who were kind and helpful and interested in my life. Friends and colleagues, such as David Smith, Paul Thacker, David Walker, Ralph Corey, Denis Spelman, and Malcolm McDonald and many others, opened doors hidden or unknown to me and enriched my life in innumerable ways. My life-long practice of setting goals each January and then critically reviewing my progress each December provided a road map and “score card” for my career; this practice helped me track progress when new opportunities arose. Lessons learned about time management and basic business principles in private practice were extremely valuable when I returned to academic life. Keeping a daily journal for the past 54 years facilitated an inner dialogue with myself that helped me keep my balance in life and stay on track in my various careers. Also, my ablity to write clearly and quickly was enhanced by making regular entries into my journal.

Using a trial-and-error process, I learned that self-education and online courses in negotiation, communication, salesmanship, leadership and writing composition provided practical and valuable skills that enhanced my enjoyment of and success in hospital epidemiology. My understanding of these skills and concepts deepened as I worked with Duke ID fellows who were interested in hospital epidemiology. As is often the case, teaching concepts to others was sometimes more educational me as the teacher than the students. Finally, my mentors, friends, colleagues, wife, and life itself taught me that one’s life work should be fun and continuously interesting. Pursuing both outcomes was, in retrospect, important to how and why I became a hospital epidemiologist.

## Advice for ID fellows who aspire to become hospital epidemiologists

Although there is no rewind button in life, detours are allowed. And, as in my case, detours may sometimes be surprisingly beneficial. I encourage all young ID specialists not to be afraid of making bold or unconventional decisions about their careers. It is perfectly fine to take chances if an unusual or interesting opportunity arises. However, regardless of whether they follow a linear or nonlinear trajectory, I urge all ID trainees to seek out and develop relationships with good mentors, to focus on being creative in your work, to set goals, and to trust your self and your ability to perservere when inevitable setbacks or bad luck or bad timing occur. For example, lack of money led to my “detour” into private practice. But I later returned to research with a new perspective and an unanticipated new set of goals. Young ID specialists should embrace their path and follow it wherever it might end. And in the end, I predict that they will find, as I did, that their unique journey was more satisfying than the destination.

